# Serratiopeptidase: An integrated View of Multifaceted Therapeutic Enzyme

**DOI:** 10.3390/biom12101468

**Published:** 2022-10-13

**Authors:** Sreelakshmi R. Nair, Subathra Devi. C

**Affiliations:** Department of Biotechnology, School of Bio Sciences and Technology, Vellore Institute of Technology, Vellore 632014, India

**Keywords:** *Serratia* sp., serratiopeptidase, anti-inflammatory, COVID-19, mucolytic, anti-biofilm, fibrinolytic

## Abstract

Microbial products have been used for the treatment of different diseases for many centuries. The serratiopeptidase enzyme provides a new hope for COVID-19-infected patients. Nowadays, anti-inflammatory drugs are easy to obtain at minimal expenditure from microbial sources. *Serratia* sp. is identified as one of the most efficient bacteria produced from serratiopeptidase. Screening for new and efficient bacterial strains from different sources has been of interest in recent years. Serratiopeptidase remains the most well-known anti-inflammatory drug of choice. Serratiopeptidase is a cheaper and safer anti-inflammatory drug alternative to NSAIDs. The multifaceted properties of serratiopeptidase may lead towards arthritis, diabetes, cancer and thrombolytic treatments. Existing serratiopeptidase treatments in combination with antibiotics are popular in the treatment of postoperative swelling. Although an exclusive number of serratiopeptidase-producing strains have been derived, there is an urge for new recombinant strains to enhance the production of the enzyme. This review explores the properties of serratiopeptidase, different therapeutic aspects, industrial production, and various analytical techniques used in enzyme recovery. In addition, the review highlights the therapeutic and clinical aspects of the serratiopeptidase enzyme to combat COVID-19-induced respiratory syndrome.

## 1. Introduction

Nowadays, enzymes are used as an alternative drug of choice to treat many ailments. De Duve [1] was the first to suggest that enzymes can be an alternative treatment for hereditary diseases. Trypsin, α-chymotrypsin, prozyme, and bromelain are the most commonly administered oral anti-inflammatory enzymes [2]. Serratiopeptidase is one of the most dominant anti-inflammatory drugs, with numerous therapeutic applications. The enzyme has anti-inflammatory, anti-biofilm, mucolytic, fibrinolytic, and wound-healing properties. The enzyme has a molecular size of 52kDa, and has the ability to bind with alpha-2-macroglobulin in blood at a ratio of 1:1 [3]. It is widely used in treating carpal tunnel syndrome, arthritis, fibrocystic breast disease, bronchitis, and sinusitis [4]. Serratiopeptidase has a strong affinity for cyclooxygenase (COX) I and II, which are crucially linked with interleukin (IL), prostaglandin (PGs), and thromboxane (TXs) production [5]. Drugs such as NSAIDs (nonsteroidal anti-inflammatory drugs), either alone or in combination with other medicines, are the most often prescribed treatment for acute inflammation [5]. They act on bonds between Arg and Gly, CysSO_3_H and Gly, Asn and Gln, Tyr and Tyr, His and Leu, Gly and Ala, Ala and Leu, Tyr and Leu, Gly and Gly, Phen and Tyr, and Tyr and Thr. This helps with reducing inflammation and controls the release of interleukins, thromboxanes, and prostaglandins. Serratiopeptidase has a long history of use as a therapeutic enzyme, and its demand in industries has been satisfied by wild, recombinant, and mutated strains. Serratiopeptidase has also been used in the treatment of Alzheimer’s disease. The enzyme has the ability to degrade amyloid plaques. In vivo studies on rat models have shown that the enzyme is capable of fighting against Alzheimer’s disease, as it helped in amyloid fibrin degradation [6]. This review is an attempt to examine and understand the available evidence regarding clinical, productive, and therapeutic aspects of serratiopeptidase. In addition, this review emphasizes the various bacterial strains used in the production of serratiopeptidase.

## 2. The Enzyme and Its Properties

Japanese researchers were the first to report and introduce the anti-inflammatory drug serratiopeptidase to the world. Enzyme formulations were created, and were widely used as medicines. After 1970, these enzyme formulations were eventually successfully marketed worldwide. The clinical studies carried out by researchers in Europe and Japan suggested serratiopeptidase as a potent anti-inflammatory drug [7,8]. Hence, the demand for enzyme began increasing worldwide. Serratiopeptidase is a metalloprotease enzyme with a molecular weight of 45–60 kDa. The enzyme contains zinc at the active site. Serratiopeptidase belongs to the group serralysin and has an EC number of 3.4.24.40. [7]. The enzyme consists of 470 amino acids which are important for its proteolytic activity. The enzyme is devoid of sulfur-containing amino acids such as cysteine and methionine. Serratiopeptidase showed maximum activity at pH 9 and 40 °C, and can be inactivated at 55 °C for 15 min [8,9].

### 2.1. Anti-Inflammatory Action of Serratiopeptidase

**Figure 1:**
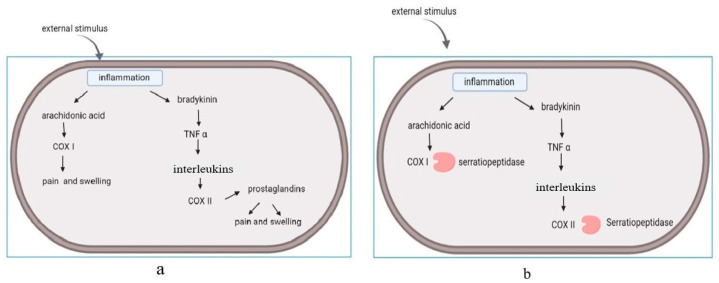
Arachidonic acid pathway: (**a**) release of interleukins and prostaglandins induce the pain and swelling. (**b**) Mode of action: serratiopeptidase acts on the cyclooxygenase enzyme (COX I and COX II) and suppresses the release of interleukins and prostaglandins.

Inflammation is an innate immune response that causes redness, swelling, and pain in the human body. It is regarded as a response of the human body against any irritant, and can be caused by many reasons, such as pathogens, injuries, and damage of cells [3]. Hence, inflammation can be regarded as a healing mechanism of our bodies to maintain homeostasis [10]. It has been observed that NSAIDs are the most commonly used drugs for inflammation [7,10]. Anti-inflammatory drugs can interact with (cyclooxygenase) COX-I and COX-II molecules. Among these enzymes, COX-I is responsible for the breakdown of arachidonic acid, which is responsible for the production of interleukins and prostaglandins [9,11]. Serine proteases are known to have a great affinity for these molecules, and can act as anti-inflammatory agents [3]. The enzymes regulated inflammatory cytokines, modified cell adhesion molecules, and acted at the site of inflammation [4,9]. In the absence of this enzyme, pain and swelling occurred at the area of injury and initiated the release of prostaglandins. (Figure 1a). This led to the onset of cascade reactions. Serratiopeptidase has the ability to bind with cyclooxygenase and suppress the release of interleukins and prostaglandins. (Figure 1b). The oral administration of serratiopeptidase tablets reduce pain and inflammation. The enzyme has its mode of action on arachidonic acid pathway (COX I and COX II), and acts on the cyclooxygenase pathway, but not on the lipooxygenase pathway (LOX). The lipooxygenase pathway (LOX) is involved in the regulation of inflammation by mediating the catalysis of SPM (specialized pro-resolving mediators) biosynthesis, and non-specific NSAID inhibition [11].

### 2.2. Wound-Healing Activity of Serratiopeptidase

In addition to the anti-inflammatory property, the enzyme also helps in wound healing. The enzyme acts by dissolving the dead tissue around the wound and hydrolyses bradykinin, serotonin, and histamine. This improves the microcirculation at the site of injury and results in wound healing [12]. There are four phases in a typical wound healing mechanism. These include the hemostasis phase, the inflammatory phase, the proliferative phase, and the maturation phase [12,13]. This enzyme can enhance microcirculation and help to maintain hemostasis [14]. Serratiopeptidase is known to reduce the capillary permeability induced by histamine, bradykinin, and serotonin, and has the ability to break the abnormal exudates and proteins as well as to improve the absorption of decomposed products through blood and lymphatics [13]. Serratiopeptidase, along with metronidazole, was found to be effective in improving wound healing in rabbits [15]. Another finding regarding serratiopeptidase was related to the tissue repair mechanism. At the site of an inflamed wound, the enzyme assisted in reducing the amount of fluids drained to the wound and facilitated microcirculation, hence improving tissue repair [13]. In a recent comparative study, the effectiveness of an enteric-coated tablet comprising fixed-dose combination (FDC) of trypsin 48 mg, bromelain 90 mg, and rutoside trihydrate 100 mg with serratiopeptidase 10 mg was observed. The results showed that serratiopeptidase was less effective than trypsin, bromelain, and rutoside trihydrate [16]. One reason for the lower efficiency may be a low dosage. A higher concentration of the drug may be more stable at gastric pH, and can facilitate the healing process.

### 2.3. Antibiofilm Activity of Serratiopeptidase

In biofilms, serratiopeptidase can alter the pathogenic phenotype of a bacterium. The use of dispersion agents may improve the effectiveness of current therapeutics. The enzymatic agents dispersin B, lysostaphin, alpha amylase, V8 protease, and serratiopeptidase were tested against methicillin-resistant and susceptible strains of *S. aureus* biofilms, both individually and in combination with vancomycin and rifampicin. When coupled with any of the dispersal agents, the effectiveness of the antibiotics was increased. Lysostaphin and serratiopeptidase were found to be the most effective dispersion agents against all of the tested strains [14]. Serratiopeptidase, a proteolytic enzyme, was originally suggested by Selan et al. [17] for the treatment of biofilm-related illnesses nearly twenty years ago. Most recently, an *S. epidermidis* (a high-slime-producing strain) infected rat model was treated with an intramuscular injection of serratiopeptidase. It was noted that 94.4% of the infected mice were recovered when compared to 62.5% in the group treated with antibiotics [18]. In the in vivo animal models, serratiopeptidase effectively acted against bacteria that produced biofilms. The antibiofilm function of enzyme may enhance the effectiveness of antibiotics in reducing *Staphylococcal* infections [18].

Another observation regarding the serratiopeptidase enzyme based on its anti-biofilm activity was against a fully matured *Staphylococcus aureus* biofilm [19]. The researchers constructed an Spep mutant by replacing the glutamic acid in the catalytic site with another amino acid (alanine), and evaluated the anti-biofilm activity of the Spep mutant. The research reports revealed that there was no proteolytic activity for the mutant strain; nevertheless, it was able to retain its anti-biofilm activity [19]. Serratiopeptidase is known to exhibit the property of modifying the adhesion molecules and thereby reducing the cell surface proteins [19]. Selan et al. [20] reported that the enzyme could alter the biofilm association of virulent strains, and that it showed activity against a completely developed biofilm. Biofilms are normally difficult to destroy. Serratiopeptidase, in combination with other antibiotics, exhibited potent anti-bioflim activity. The serratiopeptidase enzyme has reduced the expression of *Listeria monocytogens* cell surface proteins such as Ami4b, internalin B, Act A, and autolysin. The enzyme significantly precluded the adhesion of *Listeria monocytogens* in the human digestive tract [21]. According to previous reports, interestingly, it was found that the enzyme has the ability to interact only with the cell adhesion molecules that formed the biofilm. No cytotoxic activity was recorded [19,20]. The enzyme showed its effect on discrete surface proteins such as At1. It can act on these surface proteins by altering adhesins and autolysins. In a study reported by Artini et al. [22], it was stated that serratiopeptidase and carboxypeptidase showed activity against biofilm formation of different strains of *Staphylococcus aureus* and *Staphylococcus epidermidis.* The test results of the previous studies showed that only serratiopeptidase inhibited the activity of all strains. The enzyme has the ability to modify the phenotype of virulent bacteria and enhance anti-bacterial properties [22]. Another interesting fact was reported regarding the enzyme: it regulates the recruitment of immune cells to the site of inflammation [23]. The efficacy of serratiopeptidase against biofilm-forming bacteria was proven in experimental animal models. The enzyme serratiopeptidase increased the effectiveness of antibiotics in the treatment of *Staphylococcal* infections [18]. The enzyme can be supplemented with antibiotics for more effective medication.

### 2.4. Mucolytic Activity of Serratiopeptidase

Sputum production, nasal congestion, and cough are observed as some of the prevalent symptoms in COVID-19 patients. Mucolytics can increase bronchial mucus output or decrease mucus viscosity and make it easier to cough up the mucus. Serratiopeptidase may be helpful due to its caseinolytic and mucolytic effects on sputum. In patients with respiratory disorders, serratiopeptidase has improved mucociliary transportability and mucociliary clearance by lowering neutrophils and modifying the viscoelasticity of sputum [24]. Research has revealed a new combination therapy for COVID-19. A combination of vitamin D and serratiopeptidase acts as a strong mucolytic agent, and has the ability to fight against the severe effects of COVID-19 syndrome [25]. Kim et al. [26] has detailed the occurrence of other symptoms such as rhinorrhoea, hypogeusia, and nasal congestion in a large number of patients. Treatment methods such as administration of bronchodilators and mucolytic agents, along with tracheal suction, were the remedial measures for such patients [26]. Several proteolytic enzymes are known to act in a synchronized manner in the control and coordination mechanism of viral entry, viral propagation, and establishment in host cells [27]. The serratiopeptidase enzyme plays a vital role in the treatment of COVID-19 infection. Sharma et al. [28] has conferred the possibility of serratiopeptidase being used as a mucolytic drug in COVID-19 patients. It was found that serratiopeptidase can inhibit the cytokine storm in COVID-19 patients. The elevated expression of transforming growth factor (TGF-α), IL-6, and other chemokines may lead to cytokine storms in COVID-19 patients. Increased levels of IL-6 may cause acute lung disorders. This condition can be treated with medicines. Serratiopeptidase has been suggested as an effective medicine to treat the severe complications of COVID-19 [28]. Another post-COVID syndrome is cardiovascular disorder due to increased levels of D-dimers, as well as fibrin or fibrinogen products [26,29]. The cytokine storm may increase the risk of atherosclerosis and cardiac arrest. The fibrinolytic activity of serratiopeptidase, along with its proteolytic and anti-inflammatory activity, increased its potential for reducing the severity of vascular complications in COVID-19 patients [27]. Kase et al. [30] has detailed the importance of serratiopeptidase as a mucolytic agent, and compared the mucolytic activity of serratiopeptidase with seaprose. Seaprose is a proteolytic enzyme commonly used in the treatment of bronchitis. Both enzymes showed considerable mucolytic activity in the in vivo animal models.

### 2.5. Hemolytic Activity of Serratiopeptidase

The formation of blood clots in blood vessels is a major cause for cardiovascular disorders. Serine proteases are a group of enzymes that includes fibrinolytic enzymes. Serratiopeptidase, which is a serine protease, has high substrate specificity and fibrinolytic activity [31]. The enzyme serratiopeptidase has been shown to contain the property of blood clot lysis, and is able to remove arterial blocks and cysts [31]. The serine metalloprotease extracted from marine *Serratia marcescens* subsp. *sakuensis* showed efficient fibrinolytic activity [32]. Shank et al. [33] compared the hemolytic activity of both mutant and wild type *Serratia marcescens*. Mutant strains of *Serratia marcescens* exhibited hyper hemolysis. The compound serratamolide, a small cyclic amino-lipid produced by *Serratia marcescens,* was reported as an effective hemolytic and anti-microbial agent [34]. It has been observed that the swrW gene played an important role in the biosynthesis of serratamolide, also known as serrawettin [33]. Serratamolide was previously reported as a broad-spectrum antibiotic [33]. The swrW gene is responsible for the production of serratamolide. Wasserman et al. [35] reported that mutations in swrW gene expression or the hexS transcription factor gene (an inhibitor of the swrW gene) enhance the production of serratamolide. In vitro cytotoxic activity of serratamolide was reported against corneal limbal epithelial cells, as well as sheep and mouse red blood cells [35]. The compound serratamolide extracted from *Serratia marcescens* will be an effective anti-microbial and anti-cancer agent in the future.

### 2.6. Synergistic Property of Serratiopeptidase

Maheshwari et al. [36] found that the enzyme was capable of displaying a vast synergistic antimicrobial property with penicillins, fluoroquinolones, tetracycline, and cephalosporins. In combination with antibiotics, the enzyme can exhibit more intense synergistic activity in preventing biofilms [17]. Bacteria have the potential to colonise on any surface and orchestrate a coordinated response. According to reports, COX inhibitors prevent the growth of biofilms effeciently [17,37]. Previous findings have suggested that cyclooxygenase-dependent synthesis of prostaglandins is necessary for biofilm development. COX inhibitors effectively inhibited the biofilm formation when combined with aspirin, etodolac, diclofenac, celecoxib, nimesulide, ibuprofen, and meloxicam [37]. After 48 h of incubation with aspirin, etodolac along with diclofenac, which were COX-II inhibitors, showed the greatest effect, while aspirin showed 95% inhibition against biofilms [37]. Presently, researchers are focusing more intently on combination therapy to enhance the anti-inflammatory activity of serratiopeptidase. Vancomycin and rifampicin, combined with enzymatic agents such as serratiopeptidase, dispersin B, alpha-amylase, V8 protease, and lysostaphin, showed an ample amount of action against biofilms formed by methicillin-resistant susceptible strains of *S. aureus* [14] (Table 1). The efficiency and synergistic action of antibiofilms and serratiopeptidase was improved when combined with dispersal agents [14]. Serratiopeptidase is the most effective dispersion agent against most biofilm-forming bacterial strains. Table 1 represents the synergistic action of dispersal agents with different antibiotics on biofilm formation [38,39].

## 3. Enzyme Production

### 3.1. Serratia Marcescens

*Serratia marcescens* E-15 was identified as one of the potent producers of serratiopeptidase. However, the pathogenic nature of the organism has made it difficult to adapt using common production methods [40]. *Serratia* was first identified as an opportunistic human pathogen in 1959, and belongs to the Enterobacteriaceae family. It has similar characteristics to the *Klebsiella and Enterobacter* groups of bacteria [41]. Much more sophisticated and safe production strategies could make large-scale production in industries easier and more cost effective [42]. The maximum yield of enzyme production was obtained from mutant strains of *Serratia marcescens* [43]. Enhanced production of serratiopeptidase was effectively achieved by physical and chemical mutagenesis. Multiple exposures of *Serratia marcescens* to UV radiation and chemical mutagens (ethyl sulfonate) enhanced the yield and activity of the enzyme [43]. It was noted that mutant *Serratia marcescens* showed higher serratiopeptidase activity when exposed to UV and chemical mutagens. Industries have always given priority to such stable organisms. The study on thermoactive serratiopeptidase [44] from the soil isolate *Serratia marcescens* AD-W2 from India’s North-Western Himalayan area showed a specific activity of 20,492 units/mg protein with 5.28-fold purification. The molecular weight of the metalloprotease was approximately 51 kDa. At pH 9.0 and 50 °C, the purified serratiopeptidase showed maximum activity, in addition to stability over a wide range of pH values and temperatures [44]. The thermostability of the enzyme was considered to be one of the most significant properties for the large-scale industrial production.

### 3.2. Alternative Species for Production

A genetically engineered non-pathogen could be an effective replacement for much higher production of the enzyme [42]. A study conducted by Srivastava et al. [42] on recombinant expression of mature serratiopeptidase in *E. coli* resulted in failure of transformation. According to previous reports, transformed *E. coli* C_43_ (DE_3_) cells expressed proteins with lesser yield. It was also inferred that the number of transformants in pET23b(+) (without gene) and pMSrp (with mature gene) in *E. coli* DH5 was similar. There was a significant difference in the DE_3_ variant. Srivastava et al. [42] indicated that the gene had some negative effects on cells. Optimization of parameters such as nutrient composition, post induction duration, inducer concentration, and point of induction resulted in an increased expression of mature serratiopeptidase [42].

**Figure 2:**
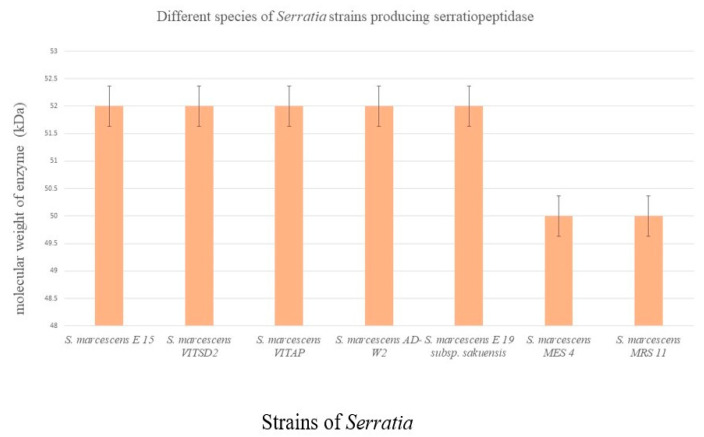
Molecular weight of serratiopeptidase produced from different strains of *Serratia*. *Serratia marcescens* E-15 [40], *Serratia marcescens* VITSD2 [48], *Serratia marcescens* VITAP [4], *Serratia marcescens* AD-W2 [44], *Serratia marcescens* subsp. *sakuensis* [32] *Serratia marcescens* MES 4 [46], *Serratia marcescens* MR 11 [46].

From silkworm gut, five different protease-producing *Serratia* strains were isolated [45]. The isolated strains are *S. indica*, *S. marcescens*, *S. piscatorum*, *S. plymuthica*, and *S. marcescens* E-15. According to reports, the E-15 strain produced the maximum amount of the enzyme compared to other strains [45]. From different species of *Serratia,* different molecular sizes of serratiopeptidase were identified (Figure 2). Koul et al. [46] identified two potent producers of serratiopeptidase. *Serratia marcescens* MES-4, an endophyte, showed 95 U/mL, and *Serratia marcescens* MRS-11, a soil isolate, showed 156 U/mL of activity [46]. Recently, recombinant expression of serratiopeptidase genes in *E. coli* was reported by Doshi et al. [47]. Fed-batch fermentation was used for the mass production of recombinant serratiopeptidase protein fusion constructs. The optimized bioreactor parameters revealed a high yield of protein and cell mass. The downstream solubilization and purification methods were also improved for the enhanced production of functional serratiopeptidase. In addition, the enzyme exhibited a novel, unanticipated self-proteolytic activity that cleaved the propeptide’s N-terminal His-SUMO fusion tag [47].

## 4. Analytical Approaches of Enzyme

Both qualitative and quantitative analysis were widely carried out for the purpose of detailed study of the serratiopeptidase enzyme, ranging from simple chemical techniques to more sophisticated chromatography methods [48]. Thin-layer chromatography was known to be a routine method for separation, and Rf values were compared with the standards. These were the most simple, quick, and easy methods used for compound identification [7,48]. High-performance liquid chromatography (HPLC) data on serratiopeptidase produced by *Serratia marcescens* VITSD2 showed a chromatogram peak of 3.45 min retention time. Sodium dodecyl sulfate-polyacrylamide gel electrophoresis (SDS—PAGE) becomes a more relevant analytical tool in identifying the molecular size of serratiopeptidase. Quantitative analysis and molecular weight determination were carried out by Ananthakrishnan et al. [49]. X-ray powder diffractometry has been applied by many researchers in order to crystallize the molecule and to understand its structure. This has relevance in identifying the molecular weight of the compound. One such dataset showed that the molecular size of serratiopeptidase was between 45,000 and 48,000 Da [7]. From the details regarding the analytical study of this enzyme, a cost-effective method may be more practical for researchers. Replacing commercially available nutrient sources with cheaper sources, as well as optimization of parameters, can increase the production rate, which, in turn, can benefit the industries. Caseinolytic assay was one of the most common methods used by most of the researchers for quantitative analysis of serratiopeptidase. Hawa et al. [50] purified and characterized serratiopeptidase from *Pseudomonas* sp., and Salamone et al. [51] quantified serratiopeptidase from *Serratia marcescens* using casein and bovine serum albumin as a substrate.

Like every pharmacological analysis, spectroscopy has been used as a valid tool in the qualitative analysis of the serratiopeptidase enzyme. Researchers have compared serratiopeptidase with other drugs and standardized the dosages for oral administration. ELISA (Enzyme linked immuno sorbent assay) is the most common assay used in the quantification of serratiopeptidase. Universally, ELISA is accepted as one of the most accurate methods for quantification. Many researchers have applied ELISA to determine the activity and concentration of serratiopeptidase [52]. Louis et al. [52] quantified serratiopeptidase produced by *Pseudomonas* sp. using ELISA. Radio immunoassays and UV microplate assays are the most sensitive methods for determining the concentration of enzymes. Even very low concentrations of the enzyme produced by different microbial strains could be detected using UV micro plate assays. When compared to other assay methods, the UV microplate method is novel, simple, fast, and specific. Sandhya et al. [53] used the UV microplate assay method for quantification of serratiopeptidase. In order to purify the enzyme, an effective, robust, and simple methodology is always needed. Pakhale et al. [54] have explained a novel strategy for purification of serratiopeptidase from *Serratia marcescens* NRRL B 23112, using an ultrasound-assisted, three-phase partitioning system [54]. According to reports, the maximum purity and recovery rate of the enzyme was obtained by the ultrasound-assisted, three-phase partitioning system when compared to three-phase partitioning (TPP). The time taken for the purification of the enzyme was dramatically reduced from 1 h to 5 min in the ultrasound-assisted, three-phase partitioning system [54]. Fuchs et al. [55] emphasized the remarkable purification efficiency of the chitin affinity chromatography method for multiple chitinolytic proteins produced from *Serratia marcescens.*

## 5. Therapeutic Aspects of Serratiopeptidase

The anti-inflammatory effects of serratiopeptidase, aspirin, trypsin, and chymotrypsin in Albino rats against carrageenan-induced paw edoema were compared by Viswanatha, Swamy, and Patil [56]. In both acute and subacute types of inflammation in rats, serratiopeptidase had superior anti-inflammatory action both on its own and in combination with aspirin. Along with a histological analysis, several inflammatory indicators, such as C-reactive protein, glutathione, myeloperoxidase, and nitric oxide, were found. When compared to the control group, serratiopeptidase decreased the disease activity index and stopped the formation of nitric oxide, as well as colonic shortening, glutathione depletion, spleen enlargement, and lipid peroxidation. Serratiopeptidase-treated mice had significantly lower C-reactive protein levels than the control mice. Moreover, the use of serratiopeptidase decreased myeloperoxidase, a significant enzyme marker of inflammation. These findings support serratiopeptidase’s ability to reduce inflammation, and thus it has been recognized as a multi-channel enzyme in terms of its wide application in treatments [57]. The enzyme has been successfully applied in atherosclerosis, in which plaques in arteries were dissolved by the proteolytic action of the enzyme. When compared with other enzymes, serratiopeptidase has been successfully used in ortholaryngiology [58]. Researchers have reported the fibrinolytic activity of serratiopeptidase and successfully used it in fibrinolytic therapy [56]. Another known application of serratiopeptidase is in dental implantation, where soft and hard gums developed inflammation upon peri implants, and anti-inflammatory enzymes were used as a treatment [58]. Serine proteases, along with other drugs, are commonly used in orthopedic medicines to treat chronic inflammation, pain, and swelling. The enzyme has great affinity with COX I and COX II, which are pain mediators [1]. An appropriate study on dosage of the enzyme must be conducted in order to control levels of the enzyme concentration in plasma, as it was found that the amount of enzymes in blood varies with body mass [59]. In 2022, it was reported that the enzyme was not able to bind with LOX or to block lipoxygenase-catalyzed biosynthesis of specialized pro-resolving mediators [60]. A pre-clinical study reported by Jadav et al. [61] indicated that serratiopeptidase was orally effective, and had anti-inflammatory activity which was equivalent to diclofenac sodium in both chronic and acute phases of inflammation. Serratiopeptidase can be used to treat osteoarthritis in combination with metformin. Ateia et al. [62] reported the impact of metformin and serratiopeptidase on knee osteoarthritis in obese patients. Metformin and serratiopeptidase combination tablets were efficient in the treatment of knee osteoarthritis. Ai-Khateeb and Nusair’s [59]. clinical study reports revealed the effect of serratiopeptidase in pain reduction, trismus, and post-operative swelling after molar surgery. Small studies in the field of dentistry, otorhinolaryngology, and orthopaedics have revealed reductions in pain and inflammation for ailments such as carpal tunnel syndrome, arthritis, and tooth extraction. Serratiopeptidase tablets have also been used in the treatment of pneumonitis, joint pain, and dermatitis. According to clinical case reports, serratiopeptidase did not show many adverse effects in treated patients [8]. Very few studies have been reported on the anti-cancer activity of serratiopeptidase. The in vitro cytotoxic activity of serratiopeptidase against colon cancer cell lines (Caco-2) was reported by Araghi et al. [63]. The findings of previous reports suggested that the enzyme has anti-cancer potential, but further in vitro and in vivo mechanistic pathway studies are needed in order to confirm the biological activity of the enzyme.

## 6. Clinical Significance

Enteric coated tablets are the most commonly available form of serratiopeptidase. Panthi et al. [64] formulated enteric coated tablets for serratiopeptidase, which exhibited persistent, stable, and significantly high drug release in the intestine. In general, glyceryl monooleate-based systems give protection to metallo-enzymes in the gastric environment. In addition, they enhanced the sustained release of the enzyme after oral administration [64]. Shah and Paradkar [65] suggested that a microenvironment-controlled, in situ, cubic phase transforming glyceryl monooleate system may give protection to serratiopeptidase as well as meticulous release. Serratiopeptidase has been used in traumatic and postoperative inflammation, laryngitis, bronchitis, expectoration of sputum in bronchial asthma, gynecology, venous inflammatory disease, cystitis, epididymitis, traumatic swelling, carpal tunnel syndrome, osteoarticular infection, sinusitis, rhinitis, and dentistry [3]. This has caused an increase in demand for the production of the enzyme, and various combinational drugs have been developed [27]. The tablets were taken orally on an empty stomach or 30 min before food. According to clinical studies, when compared to methylprednisolone, serratiopeptidase showed low analgesic action and efficient management of edema and trismus [66]. The oral administration of this enzyme can reduce inflammation and pain in AIDS, as well as in hepatitis B & C infections [67]. This led to an increase in the use of serratiopeptidase in the field of medicine. Cancer nanomedicine has created a revolution in the field of medicine [68]. Serratiopeptidase in combination with nanodrug delivery systems has been an emerging technology in cancer therapy. Anti-inflammatory agents such as serratiopeptidase may help in overcoming the adverse effects of anti-cancer agents. Jaiswal and Mishra [69] reported that the co-delivery of curcumin and serratiopeptidase along with nanoparticles showed enhanced anti-cancer activity against HeLa and MCF-7 cells. Serratiopeptidase can be viewed as a viable competitor in contemporary medicine. Hence, the synergistic activity of serratiopeptidase has a vital role in emphasizing its clinical importance [17,38,39].

## 7. Conclusions

The proteolytic enzyme serratiopeptidase has an enormous number of therapeutic applications and significant analytical importance. Anti-inflammatory, anti-biofilm, mucolytic, and synergistic action are the potential targets of drug therapy. Being a mucolytic agent, serratiopeptidase has been used in treatment of the COVID-19 infection. Among these studies, the serratiopeptidase was known to exhibit significant synergistic activity with various antibiotics in resolving infection and inflammation. Serratiopeptidase has fibrinolytic and anti-cancer activity. In the future, there will be a great demand for the enzyme due to its multifaceted properties. Researchers are also focusing on serratiopeptidase nanoparticles for the purpose of targeted delivery and restricted action on the selected sites. Most researchers emphasize the synergistic action of serratiopeptidase in the treatment of arthritis, diabetes, and Alzheimer’s disease. The current review explores the different agents involved in the industrial production of serratiopeptidase. So far, very few studies have been conducted on serratiopeptidase coding genes. Hence, a more detailed study on the genome of *Serratia* species will lead to the development of new and potent strains for large-scale production. Gene cloning and vector therapy will be helpful for industrialists to enhance the production rate of the enzyme. Nowadays, pharmaceutical companies are targeting serratiopeptidase production due to its multifaceted properties. Pharmaceutical companies are focused on the construction of recombinant strains to enhance the yield and purity of the enzyme. Serratiopeptidase is a miracle enzyme which has the potential to replace NSAIDs. With this multifaceted value, this enzyme could be a pioneer in the treatment of COVID-19 and other infectious diseases.

## Figures and Tables

**Table 1:** Synergistic property of enzyme with different antibiotics.

Sl No	Antibiotics	Effect of Enzymes	References
1.	Ofloxacin	Enhanced the activity of ofloxacin and inhibited biofilm formation.	[17]
2.	Azithromycin	Effective against different strains of biofilm forming *Staphylococcus* sp.	[38]
3.	Levofloxacin	Eradicated > 90% of the preformed biofilm.	[39]
4.	Vancomycin and rifampicin	Effective in dispersing most of the biofilm forming bacteria	[14]

## Data Availability

Not applicable.

## References

[1] De Duve C. (1966). The significance of lysosomes in pathology and medicine. Proc. Inst. Med. Chic..

[2] Tasaka K., Meshi T., Akagi M., Kakimoto M., Saito R., Okada I., Maki K. (1980). Anti-Inflammatory Activity of a Proteolytic Enzyme, Prozime-10. Pharmacology.

[3] Devi C.S., Elizabeth J.R., Saravanan H., Naine S.J., Srinivansan V.M. (2013). Screening and molecular characterization of Serratia mar-cescens VITSD2: A strain producing optimum serratiopeptidase. Front. Biol..

[4] Mohankumar A., Raj R.K. (2011). Production and Characterization of Serratiopeptidase Enzyme from Serratia Marcescens. Int. J. Biol..

[5] Steiger S., Harper J.L. (2013). Mechanisms of Spontaneous Resolution of Acute Gouty Inflammation. Curr. Rheumatol. Rep..

[6] Metkar S.K., Girigoswami A., Vijayashree R., Girigoswami K. (2020). Attenuation of subcutaneous insulin induced amyloid mass in vivo using Lumbrokinase and Serratiopeptidase. Int. J. Biol. Macromol..

[7] Maeda H., Morihara K. (1995). [24] Serralysin and related bacterial proteinases. Methods Enzymol..

[8] Gupte V., Luthra U. (2017). Analytical techniques for serratiopeptidase: A review. J. Pharm. Anal..

[9] Bhagat S., Agarwal M., Roy V. (2013). Serratiopeptidase: A systematic review of the existing evidence. Int. J. Surg..

[10] Suma K.C., Manasa H., Likhitha A., Nagamani T.S. (2020). Isolation, Purification, and Characterization of Serratiopeptidase Enzyme from *Serratia marcescens*. Int. J. Innov. Sci. Res. Technol..

[11] Srivastava S., Singh D., Patel S., Singh M.R. (2017). Treatment of rheumatoid arthritis by targeting macrophages through folic acid tailored superoxide dismutase and serratiopeptidase. J. Drug Deliv. Sci. Technol..

[12] Teller P., White T.K. (2011). The Physiology of Wound Healing: Injury Through Maturation. Perioper. Nurs. Clin..

[13] Strodtbeck F. (2001). Physiology of wound healing. Newborn Infant Nurs. Rev..

[14] Hogan S., Zapotoczna M., Stevens N., Humphreys H., O’Gara J., O’Neill E. (2017). Potential use of targeted enzymatic agents in the treatment of Staphylococcus aureus biofilm-related infections. J. Hosp. Infect..

[15] Rath G., Johal E.S., Goyal A.K. (2010). Development of Serratiopeptidase and Metronidazole Based Alginate Microspheres for Wound Healing. Artif. Cells Blood Substit. Biotechnol..

[16] Mungantiwar A., Bhatt N., Shrivastava P., More J., Shaikh R. (2021). A Randomized, Open-Label, Phase IV Clinical Study to Compare the Safety and Efficacy of the Fixed-Dose Combination of Trypsin, Bromelain, and Rutoside versus Serratiopeptidase in Minor Sur-gical Wound. IJRAMT..

[17] Selan L., Berlutti F., Passariello C., Comodi-Ballanti M.R., Thaller M.C. (1993). Proteolytic enzymes: A new treatment strategy for prosthetic infections?. Antimicrob. Agents Chemother..

[18] Mecikoglu M., Saygi B., Yildirim Y., Karadag-Saygi E., Ramadan S.S., Esemenli T. (2006). The effect of proteolytic enzyme serratiopep-tidase in the treatment of experimental implant-related infection. JBJS..

[19] Selan L., Papa R., Tilotta M., Vrenna G., Carpentieri A., Amoresano A., Pucci P., Artini M. (2015). Serratiopeptidase: A well-known metallo-protease with a new non-proteolytic activity against *S. aureus* biofilm. BMC Microbiol..

[20] Selan L., Artini M., Papa R., Dhanasekaran D. (2016). Compounds from natural sources for new diagnostics and drugs against biofilm infections. Microbial Biofilms—Importance and Applications.

[21] Longhi C., Scoarughi G.L., Poggiali F., Cellini A., Carpentieri A., Seganti L., Pucci P., Amoresano A., Cocconcelli P.S., Artini M. (2008). Protease treatment affects both invasion ability and biofilm formation in Listeria monocytogenes. Microb. Pathog..

[22] Artini M., Papa R., Scoarughi G.L., Galano E., Barbato G., Pucci P., Selan L. (2012). Comparison of the action of different proteases on virulence properties related to the staphylococcal surface. J. Appl. Microbiol..

[23] Tiwari M. (2017). The role of serratiopeptidase in the resolution of inflammation. Asian J. Pharm. Sci..

[24] Gioia M., Ciaccio C., Calligari P., De Simone G., Sbardella D., Tundo G., Fasciglione G.F., Di Masi A., Di Pierro D., Bocedi A. (2020). Role of proteolytic enzymes in the COVID-19 infection and promising therapeutic approaches. Biochem. Pharmacol..

[25] Gupta K.K., Rahman A., Kumar A., Gavel P., Asia P. (2021). Adjuvant therapy with Serratiopeptidase and Vitamin D for COVID-19 patients: A new perspective. Int. J. Med. Sci..

[26] Kim G.-U., Kim M.-J., Ra S., Lee J., Bae S., Jung J., Kim S.-H. (2020). Clinical characteristics of asymptomatic and symptomatic patients with mild COVID-19. Clin. Microbiol. Infect..

[27] Farooqi F.I., Morgan R.C., Dhawan N., Dinh J., Yatzkan G., Michel G. (2020). Airway Hygiene in COVID-19 Pneumonia: Treatment Responses of 3 Critically Ill Cruise Ship Employees. Am. J. Case Rep..

[28] Sharma C., Jha N.K., Meeran M.N., Patil C.R., Goyal S.N., Ojha S. (2021). Serratiopeptidase, a serine protease anti-inflammatory, fibrinolytic, and mucolytic drug can be a useful adjuvant for management in COVID-19. Front. Pharmacol..

[29] Connors J.M., Levy J.H. (2020). COVID-19 and its implications for thrombosis and anticoagulation. Blood.

[30] Kase Y., Seo H., Oyama Y., Sakata M., Tomoda K., Takahama K., Hitoshi T., Okano Y., Miyata T. (1982). A new method for evaluating mucolytic expectorant activity and its application. II. Application to two proteolytic enzymes, serratiopeptidase and seaprose. Arzneim.-Forsch..

[31] Kotb E. (2013). Activity assessment of microbial fibrinolytic enzymes. Appl. Microbiol. Biotechnol..

[32] Krishnamurthy A., Belur P.D. (2018). A novel fibrinolytic serine metalloprotease from the marine Serratia marcescens subsp. sakuensis: Purification and characterization. Int. J. Biol. Macromol..

[33] Shanks R.M.Q., Stella N.A., Lahr R.M., Wang S., Veverka T.I., Kowalski R.P., Liu X. (2012). Serratamolide is a Hemolytic Factor Produced by Serratia marcescens. PLoS ONE.

[34] Shanks R.M., Lahr R.M., Stella N.A., Arena K.E., Brothers K.M., Kwak D.H., Liu X., Kalivoda E.J. (2013). A Serratia marcescens PigP homolog controls prodigiosin biosynthesis, swarming motility and hemolysis and is regulated by cAMP-CRP and HexS. PLoS ONE.

[35] Wasserman H.H., Keggi J.J., McKeon J.E. (1962). The structure of Serratamolide1-3. J. Am. Chem. Soc..

[36] Maheshwari M., Miglani G., Mali A., Paradkar A., Yamamura S., Kadam S. (2006). Development of tetracycline-serratiopeptidase-containing periodontal gel: Formulation and preliminary clinical study. AAPS PharmSciTech.

[37] Alem M.A.S., Douglas L.J. (2004). Effects of Aspirin and Other Nonsteroidal Anti-Inflammatory Drugs on Biofilms and Planktonic Cells of *Candida albicans*. Antimicrob. Agents Chemother..

[38] Thaller M., Selan L., Fiorani P., Passariello C., Rizzo L., Speziale F. (1997). A comparative in vitro evaluation of different therapeutic protocols for vascular graft infections. Eur. J. Vasc. Endovasc. Surg..

[39] Gupta P.V., Nirwane A.M., Belubbi T., Nagarsenker M.S. (2017). Pulmonary delivery of synergistic combination of fluoroquinolone antibiotic complemented with proteolytic enzyme: A novel antimicrobial and antibiofilm strategy. Nanomed. Nanotechnol. Biol. Med..

[40] Anil C.S., Kashinath M.A. (2013). Production, characterization & optimization of potent protease (serratiopeptidase) from Serratia mar-cescense 15. Int. J. Pharm. Res. Allied Sci..

[41] Acar J.F. (1986). Serratia marcescens Infections. Infect. Control Hosp. Epidemiol..

[42] Srivastava V., Mishra S., Chaudhuri T.K. (2019). Enhanced production of recombinant serratiopeptidase in Escherichia coli and its charac-terization as a potential biosimilar to native biotherapeutic counterpart. Microb. Cell Factories.

[43] Gopinath S., Venkataprasad R., Rajnish K.N., Datta S., Selvarajan E. (2020). Enhancement of serrapeptase hyper producing mutant by com-bined chemical and UV mutagenesis and its potential for fibrinolytic activity. J. Pure Appl. Microbiol..

[44] Chander D., Khosla J.K., Koul D., Hossain M., Dar M.J., Chaubey A. (2021). Purification and characterization of thermoactive serrati-opeptidase from Serratia marcescens AD-W2. AMB Express.

[45] Barman S., Bhattacharya S.S., Mandal N.C. (2020). Serratia. Benef. Microbes Agro-Ecol..

[46] Koul D., Chander D., Manhas R.S., Chaubey A. (2020). Isolation and Characterization of Serratiopeptidase Producing Bacteria from Mulberry Phyllosphere. Curr. Microbiol..

[47] Doshi P., Dantroliya S., Modi A., Shukla A., Patel D., Joshi C., Joshi M. (2022). Enhanced Production Process of Recombinant Mature Serra-ti-opeptidase in Escherichia coli Using Fed-Batch Culture by Self-Proteolytic Activity of Fusion Protein. Fermentation.

[48] Kyoko T., Keiko M., Kayoko S. (1981). Quality tests of high-molecular-weight substances by chromatography. Jpn. J. Relig. Stud..

[49] AAnanthakrishnan B., Ramesh M.S. (2013). Muthuraman, Optimization studies in the production and purification of serratiopeptidase from Serratia marcescens UV mutant SM3. Int. J. Pharm. Pharm. Sci..

[50] El-Hawa M. (1997). Abou, Purification and characterization of protease produced by *Pseudomonas aeruginosa*. Egypt. J. Microbiol..

[51] Salamone P.R., Wodzinski R.J. (1997). Production, purification and characterization of a 50-kDa extracellular metalloprotease from Serratia marcescens. Appl. Microbiol. Biotechnol..

[52] Louis D., Bernillon J., Païsse J.O., Wallach J.M. (1999). Use of liquid chromatography-mass spectrometry coupling for monitoring the serra-lysin-catalyzed hydrolysis of a peptide library. J. Chromatogr..

[53] Sandhya K.V., Devi S.G., Mathew S.T. (2008). Quantitation of serrapeptase in formulations by UV method in the microplate format. Curr. Drug Deliv..

[54] Pakhale S.V., Bhagwat S.S. (2016). Purification of serratiopeptidase from Serratia marcescens NRRL B 23112 using ultrasound assisted three phase partitioning. Ultrason. Sonochemistry.

[55] Fuchs R.L., McPherson S.A., Drahos D.J. (1986). Cloning of a *Serratia marcescens* Gene Encoding Chitinase. Appl. Environ. Microbiol..

[56] Swamy A.V., Patil P. (2008). Effect of some clinically used proteolytic enzymes on inflammation in rats. Indian J. Pharm. Sci..

[57] Rajinikanth B., Venkatachalam V.V., Manavalan R. (2014). Investigations on the potential of serratiopeptidase—A proteolytic enzyme, on acetic acid induced ulcerative colitis in mice. Int. J. Pharm. Pharm. Sci..

[58] Jadhav S.B., Shah N., Rathi A., Rathi V., Rathi A. (2020). Serratiopeptidase: Insights into the therapeutic applications. Biotechnol. Rep..

[59] Al-Khateeb T., Nusair Y. (2008). Effect of the proteolytic enzyme serrapeptase on swelling, pain and trismus after surgical extraction of mandibular third molars. Int. J. Oral Maxillofac. Surg..

[60] Luthra U., Babu P., Patel Y., Ramesh J.V., Sharma M., Majeed I., Subbiah S.K., Pandiyan R. (2022). Serratiopeptidase: A statistical approach towards enhancement of fermentation and biomass product recovery. Biomass-Convers. Biorefinery.

[61] Jadav S.P., Patel N.H., Shah T.G., Gajera M.V., Trivedi H.R., Shah B.K. (2010). Comparison of anti-inflammatory activity of serratiopeptidase and diclofenac in albino rats. J. Pharmacol. Pharmacother..

[62] Ateia Y.A., Al-Edanni M.S., Al-Qurtas M.I. (2018). Impact of metformin and serratiopeptidase in obese patients with knee osteoarthritis. Int. J. Pharm. Pharm. Sci..

[63] Araghi A., Hashemi S., Sepahi A.A., Faramarzi M.A., Amin M. (2019). Purification and study of anti-cancer effects of Serratia mar-cescens ser-ralysin. Iran J. Microbiol..

[64] Panthi V.K., Jha S.K., Chaubey R., Pangeni R. (2021). Formulation and development of Serratiopeptidase enteric coated tablets and analytical method validation by UV Spectroscopy. Int. J. Anal. Chem..

[65] Shah M.H., Paradkar A. (2005). Cubic liquid crystalline glyceryl monooleate matrices for oral delivery of enzyme. Int. J. Pharm..

[66] Chappi D.M., Suresh K.V., Patil M.R., Desai R., Tauro D.P., Bharani K.N.S.S., Parkar M.I., Babaji H.V. (2015). Comparison of clinical efficacy of methylprednisolone and serratiopeptidase for reduction of postoperative sequelae after lower third molar surgery. J. Clin. Exp. Dent..

[67] Chanalia P., Gandhi D., Jodha D., Singh J. (2011). Applications of microbial proteases in pharmaceutical industry: An overview. RRMM..

[68] Shi J., Kantoff P.W., Wooster R., Farokhzad O.C. (2017). Cancer nanomedicine: Progress, challenges and opportunities. Nat. Rev. Cancer.

[69] Jaiswal S., Mishra P. (2018). Co-delivery of curcumin and serratiopeptidase in HeLa and MCF-7 cells through nanoparticles show improved anti-cancer activity. Mater. Sci. Eng. C.

